# Pifithrin-α ameliorates glycerol induced rhabdomyolysis and acute kidney injury by reducing p53 activation

**DOI:** 10.1080/0886022X.2022.2048857

**Published:** 2022-03-13

**Authors:** Chen Yuqiang, Zhang Lisha, Wen Jiejun, Xue Qin, Wang Niansong

**Affiliations:** a Department of Nephrology, Shanghai Jiao Tong University Affiliated Sixth People’s Hospital, Shanghai, China; b Department of Emergency, Shanghai Punan Hospital, Pudong New District, Shanghai, China

**Keywords:** Rhabdomyolysis, acute kidney injury, pifithrin-α, oxidative stress

## Abstract

**Objectives:**

Rhabdomyolysis is a series of symptoms caused by the dissolution of striped muscle, and acute kidney injury (AKI) is a potential complication of severe rhabdomyolysis. The underlying causes of AKI are remarkably complex and diverse. Here, we aim to investigate whether pifithrin-α protected against rhabdomyolysis-induced AKI and to determine the involved mechanisms.

**Methods:**

Intramuscular injection in the right thigh caudal muscle of C57BL/6J mice with 7.5 ml/kg saline (Group A) or of the same volume 50% glycerol was used to induce rhabdomyolysis and subsequent AKI (Group B). Pifithrin-α was injected intraperitoneally 4 h before (Group C) or 4 h after (Group D) the glycerol injection. Serum creatine kinase, blood urea nitrogen, and creatinine were determined, and the renal cortex was histologically analyzed. Renal expression levels of interested mRNAs and proteins were determined and compared, too.

**Results:**

Intramuscular injection of glycerol induced rhabdomyolysis and subsequent AKI in mice (Groups B–D). Renal function reduction and histologic injury of renal tubular epithelial cells were associated with increased p53 activation, oxidative stress, and inflammation. Notably, compared with pifithrin-α rescue therapy (Group D), pretreatment of pifithrin-α (Group C) protected the mice from severe injury more effectively.

**Conclusions:**

Our present study suggests that p53 may be a therapeutic target of AKI caused by glycerol, and the inhibition of p53 can block glycerol-mediated AKI by using pharmacological agents instead of genetic inhibitory approaches, which further supports that p53 played a pivotal role in renal tubular injury when challenged with glycerol.

## 1. Introduction

Rhabdomyolysis is a series of symptoms caused by the dissolution of striped muscle. It is characterized by the leakage of muscle-cell contents such as electrolytes, myoglobin, creatine kinase, aldolase, lactate dehydrogenase, alanine aminotransferase, and aspartate aminotransferase into the blood circulation. Massive muscle necrosis, limb weakness, myalgia, swelling, and gross pigmenturia without hematuria, are the common denominator of rhabdomyolysis [1]. Acute kidney injury (AKI) can be a potential complication of severe rhabdomyolysis, regardless of the causes of rhabdomyolysis, and the prognosis is substantially worse if AKI develops, with high rates of mortality and increased risk of chronic kidney diseases (CKD) [2,3]. Many studies showed rhabdomyolysis could be induced by different conditions including metabolism disorders, trauma, drugs, toxins, and infections. As a complication of rhabdomyolysis, AKI is quite common.

The pathogenesis of AKI is thought to be initiated by systemic and localized stress conditions that cause toxic, hypoxic, and inflammatory insults to renal tubular epithelial cells (RTECs), followed by incomplete repair and maladaptive cellular responses [4]. Thus, targeting pathways involved in RTEC cell death and dysfunction could be an effective strategy to prevent acute renal injury and subsequent AKI-to-CKD transition [5]. It has been established that endoplasmic reticulum (ER) stress-mediated apoptosis of tubular epithelium cells played crucial roles in rhabdomyolysis-induced AKI [6]. Organelle-mediated stress, particularly ER stress, has recently emerged as a major pathophysiological paradigm underlying AKI including renal tubular cell apoptosis, inflammatory response, macrophage infiltration, and oxidative stress [7,8]. As the detailed mechanisms have not been fully comprehended, studies on the pathophysiological characteristics are greatly needed to acquire effective management of AKI associated with rhabdomyolysis.

Generally, p53 is a tumor suppressor and can be induced by cancer and cellular stress in normal cells. Interestingly, AKI was also associated with the upregulation of several known p53 target genes, including Bax and p21, and this association was attenuated in p53-KO mice [9,10]. Nevertheless, the pathogenic role of p53 in AKI remains controversial and the underlying mechanism is unclear [10–13]. Pifithrin-α is a specific p53 inhibitor and it can facilitate the progression of RTECs through the G2/M phase, protecting them against injury [14]. Tubular-specific ablation of p53 in mice or pifithrin-α-mediated inactivation of p53 also reduces the secretion of fibrotic effectors and attenuates the transition from acute to chronic renal injury, further supporting the involvement of p53 in disease progression [15]. Moreover, blockade of p53 by pifithrin-a, siRNA, or proximal tubule–targeted gene ablation reduced ischemic AKI in diabetic mice [13]. Furthermore, recent studies showed that p53 activation controls long-term outcomes of AKI [12].

Taken together, mechanisms in rhabdomyolysis-induced AKI still need further studies. In this study, we aim to investigate whether pifithrin-α protected against rhabdomyolysis-induced AKI and to determine the possible mechanisms.

## 2. Method

### 2.1. Animal model

Male C57BL/6J mice aged 10 weeks old were purchased from Shanghai SLAC Laboratory Animal Co., Ltd (Shanghai, China) and housed in a pathogen-free, temperature-controlled environment with a 12-h/12-h light/dark photocycle. Animals had free access to food and tap water to avoid dehydration-related hypovolemia. All reported experiments were approved by a local animal care and use committee. Similar to the pioneer studies [16,17], the animals were intramuscularly injected in the right thigh caudal muscle with 7.5 ml/kg 50% glycerol (Fontenay sous Bois, France) to induce rhabdomyolysis and AKI or saline of the same volume as a control. To study the effect of p53 inhibitor pifithrin-α (PFT-α, purchased from Sigma, St. Louis, MO, USA), the mice were allocated into four groups: mice in Group A were intramuscularly injected with normal saline at 0 h and served as control. Mice in Group B were intramuscularly injected with glycerol and served as AKI model without pifithrin-α treatment. Mice in Group C were intramuscularly injected with glycerol and intraperitoneal injection of pifithrin-α (4.4 mg per kg body weight) was given 4 h before AKI induction. Mice in Group D were intramuscularly injected with glycerol and intraperitoneal injection of pifithrin-α (4.4 mg per kg body weight) was given 4 h after AKI induction.

### 2.2. Renal function

Blood was drawn from the mouse-tail vein and serum was collected to measure blood-urea nitrogen (BUN) and creatinine by using a Pentra 400 analyzer (Horiba Medical, Grabels, France). Serum creatine kinase was determined by the hospital’s blood automatic biochemical analyzer in the central laboratory.

### 2.3. RNA isolation and quantitative real-time polymerase chain reaction (qRT-PCR)

Total RNA was extracted from the right renal cortex using TRIzol reagent (Invitrogen, Grand Island, NY, USA) according to the manufacturer’s protocol. RNA concentrations were measured using the SpectraMax microplate spectrophotometer (Molecular Devices, Sunnyvale, CA, USA). For reverse transcription, 1.0 µl of cDNA and SYBR-Green real-time PCR Master Mix (Takara Co., Ltd., Tokyo, Japan) were used according to the manufacturer’s protocol. The PCR amplifications were performed in a 96-well plate for 1 cycle of 94 °C for 30 s, 40 cycles of 95 °C for 5 s, and 60 °C for 30 s on Applied Biosystems 7900HT. The expression level was analyzed by SDS2.4 software (Applied Biosystems) and internally normalized to GAPDH with the 2^–ΔΔ^Ct method. The primers used for qRT-PCR were listed as below: GAPDH forward 5′-AACTTTGGCATTGTGGAAGG, reverse 5′-ACACATTGGGGGTAGGAACA; GRP78 forward 5′-CATGGTTCTCACTAAAATGAAGG, reverse 5′-GCTGGTACAGTAACAACTG; CHOP forward 5′-AGCTGGAAGCCTGGTATGAGGA, reverse 5′-AGCTAGGGACGCAGGGTCAA; IL-6 forward 5′-AACGATGATGCACTTGCAGA, reverse 5′-TGGTACTCCAGAAGACCAGAGG.

### 2.4. Western blot analysis

Total protein samples were extracted from the right renal cortex using lysis buffer containing protease inhibitor. The protein concentrations were measured using the Bio-Rad protein assay system (Bio-Rad Laboratories, Hercules, CA, USA). After boiling the samples for 5 min, the protein samples were run on SDS-PAGE (polyacrylamide gels). The lysates were resolved by electrophoresis (70 V for 25 min and 120 V for 1.5 h) and transferred onto NC membranes (nitrocellulose membrane; Bio-Rad Laboratories). After blocking, the NC membranes were treated overnight at 4 °C with the following primary antibodies: C/EBP homologous protein (CHOP; catalog no. 2895), GRP78 (catalog no. 3177), total-p53(catalog no. 9282) and Phospho-p53 (Ser15) (catalog no. 9284), and GAPDH (catalog no. 2118) were from Cell Signaling Technology. The density for each tested protein was normalized against GAPDH. Western blot bands were quantified using Odyssey v1.2 software by measuring the band intensity (Area x OD; Optical Density) for each group. All the presented results were representative of at least three independent experiments.

### 2.5. Histologic analyses

Periodic Acid-Schiff coloration was performed using Hematoxylin (S3309; Dako, Trappes, France), Periodic acid (198401000; Thermo Fisher Scientific, Geel, Belgium), and Schiff reagent (109033; EMD Millipore, Darmstadt, Germany). Mouse tissues were first de-waxed in toluene and rehydrated through a series of graded ethanol washes before endogenous peroxidase blockage.

### 2.6. Immunohistochemical analyses

Standard histochemical staining procedures were followed as described anywhere else. Specific primary antibodies were incubated on mouse tissue sections for the detection of Kidney injury molecule-1 (KIM-1, R&D systems, catalog no. AF1817). Negative controls for the immunohistochemical procedures included substitution of the primary antibody with non-immune sera. To evaluate the tubulointerstitial injury detected by KIM-1, 10 cortical fields of each slide were examined. At least 10 renal cortical fields of each slide were examined, and the kidney sections were systematically scored as described in references [18,19], based on the percentage of injured tubules: 0, none; 1, <25%; 2, 26%–45%; 3, 51%–75%; and 4, >75%.

### 2.7. Renal apoptosis assay

Renal apoptosis was examined *in situ* by TUNEL assay kit from Roche Applied Science (Indianapolis, IN, USA). Briefly, paraffin-embedded renal tissue sections were deparaffinized and permeabilized by 30 min of incubation at 37 °C in 0.1 mol/l sodium citrate (pH 6.0). The sections were then exposed to the TUNEL reaction mixture containing green-labeled dUTP. TUNEL-positive nuclei were identified by fluorescence microscopy.

### 2.8. Statistical analyses

Normally distributed data obtained from this study were expressed as means ± SEM. Statistical analyses were performed using a two-tailed t-test or one-way ANOVA followed by the Newman-Keuls as *post hoc* tests. For nonparametric or mixed data, Kruskal-Wallis test was used where applicable in GraphPad Prism 9.0 (GraphPad Software, San Diego, CA, USA).

## 3. Results

### 3.1. Intramuscular injection of glycerol induced rhabdomyolysis and AKI in mice

**Figure 1.**
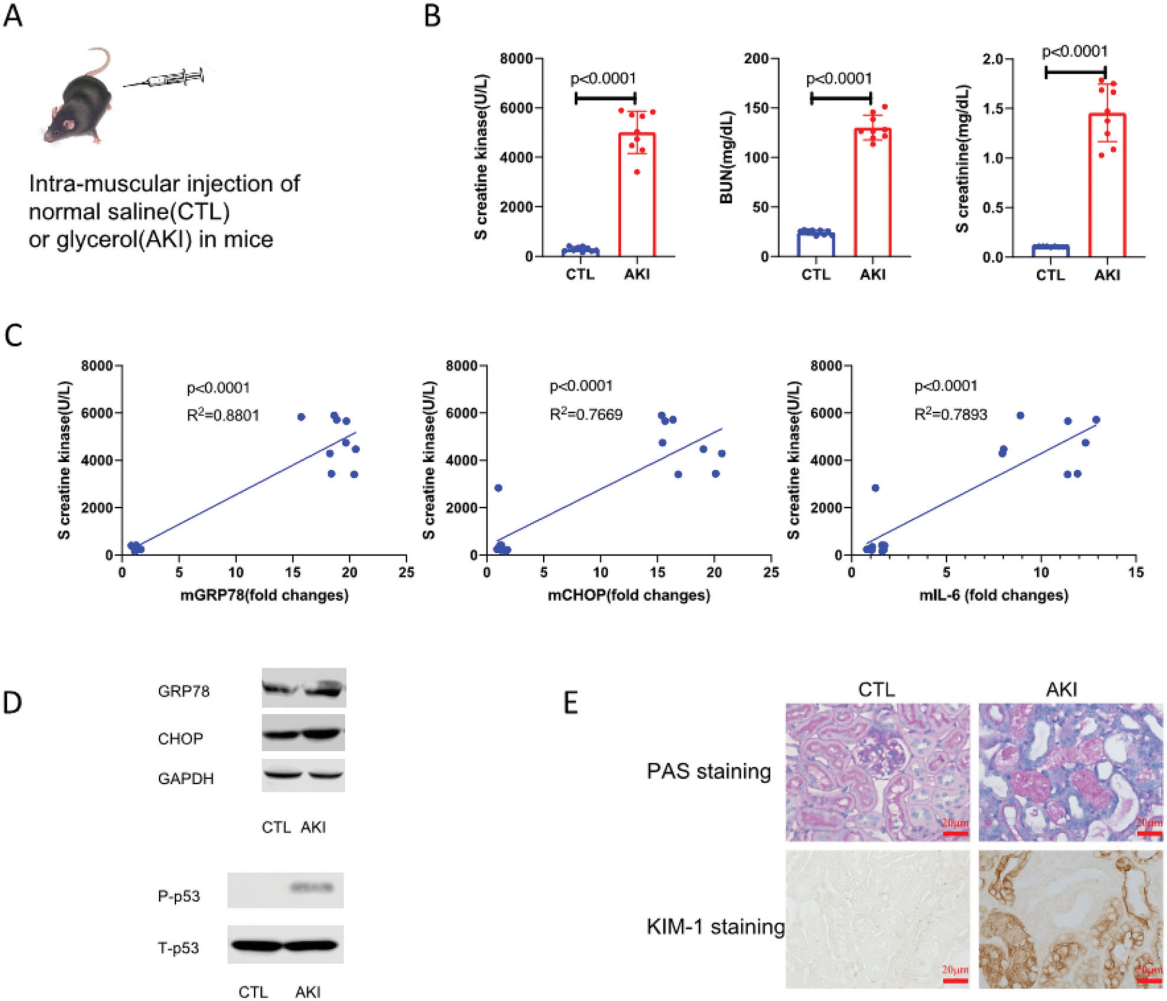
Intramuscular injection of glycerol induced rhabdomyolysis and rhabdomyolysis associated AKI. (A) Male C57BL/6J mice were intramuscularly injected in right thigh caudal muscle with 7.5 ml/kg 50% glycerol, thereafter, the mice underwent excessively swollen thigh caudal muscle. (B) Levels of serum creatine kinase, BUN, and creatinine elevated greatly in glycerol injected mice compared with control (*p* < 0.001). (C) The correlation analysis showed that the level of creatine kinase was positively correlated with renal mGRP78, mCHOP, and mIL-6, respectively (*p* < 0.001). (D) Western blot results showed that protein levels of GRP78, CHOP, and phosphorylated p53(Ser15) in the renal cortex in each AKI mice induced by rhabdomyolysis were enhanced significantly. All the presented results were representative of at least three independent experiments. (E) Twenty-four hours after injection, the mice were sacrificed, and the right kidneys were harvested for PAS staining and KIM-1 immune histologic analysis (×400).

Results showed that 24 h after glycerol injection, the mice underwent excessively swollen thigh caudal muscle and classic AKI (Figure 1(A)). Beyond the clinical manifestations, the elevated level of creatine kinase, BUN, and creatinine also demonstrated that intramuscular injection of glycerol induced rhabdomyolysis and rhabdomyolysis associated with AKI successfully in mice (Figure 1(B)).

### 3.2. Glycerol induced rhabdomyolysis and related AKI is associated with increased oxidative stress, inflammation, and p53 activation

We determined mRNA levels of oxidative stress-related genes such as C/EBP homologous protein (CHOP), glucose-regulated protein (GRP78), and inflammation-related gene interleukin-6 (IL-6), protein levels of the total p53 and phospho-p53 (Ser15). Seral creatine kinase levels of mice were positively correlated with levels of mGRP78, mCHOP, and mIL-6 in the renal cortex, respectively (Figure 1(C), *p* < 0.001). Further, protein levels of GRP78 and CHOP in renal cortex in AKI mice induced by glycerol were increased significantly compared with AKI-free controls. Meanwhile, phospho-p53 (Ser15) levels in AKI mice were significantly enhanced compared with controls (Figure 1(D)). All those data indicated that the increased oxidative stress and inflammation were of importance in glycerol-induced rhabdomyolysis and AKI. Meanwhile, p53 activation (phosphorylation) should play a role in glycerol-induced rhabdomyolysis and AKI. PAS staining revealed massive inflammatory cells infiltrated in the tubulointerstitium, enlarged renal tubular lumen, part of the basement membrane of RTECs shed, and the brush border destroyed or even vanished. Further, the KIM-1 staining outlined the injured RTECs distinctively in our AKI model (Figure 1(E)).

### 3.3. Pretreatment of pifithrin-α is superior to pifithrin-α rescue therapy in ameliorating glycerol induced rhabdomyolysis and AKI

**Figure 2.**
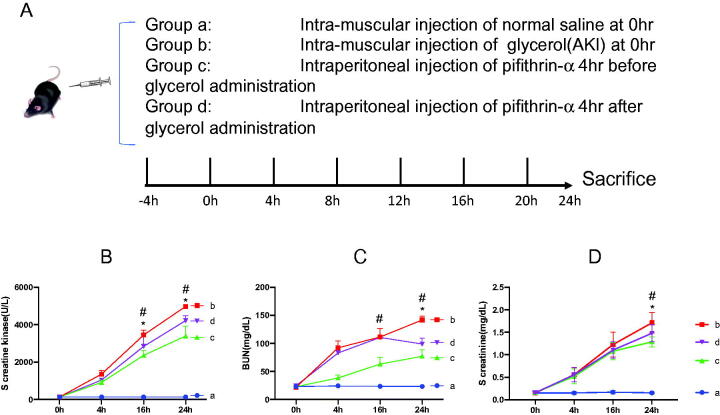
Serum creatine kinase and renal function parameters in glycerol and pifithrin-α treated mice. (A) Grouping of the mice. In each group, the number of mice was no less than five. (B) Serum creatine kinase at every checkpoint in each group was determined and compared, respectively. (C and D) Serum BUN and creatinine levels at every checkpoint in each group were determined and compared, respectively. *p < 0.05 when Group C compared with Group D; #*p* < 0.001 when Group C compared with Group B.

To explore whether p53 inhibitor pifithrin-α has a protective role in glycerol-induced rhabdomyolysis and AKI, the mice were allocated into four groups (Figure 2(A)). Serum creatine kinase, BUN, and creatinine at every checkpoint in each group were determined and compared, respectively (Figure 2(B) through 2(D)). Interestingly, levels of serum creatine kinase in the Groups B, C, and D all increased in a time-dependent manner. Moreover, 16 h after glycerol induction, levels of serum creatine kinase in the pifithrin-α injected group began to show a moderated uptrend compared to that in the Group B (*p* < 0.05), which indicated that pifithrin-α may protect mice from severe rhabdomyolysis. Beyond that, pretreatment of pifithrin-α was superior to pifithrin-α rescue therapy in ameliorating glycerol-induced rhabdomyolysis for its creatine kinase level was the lowest in all mice with rhabdomyolysis (Figure 2(B)). Similarly, pretreatment of pifithrin-α was superior to pifithrin-α rescue therapy in ameliorating glycerol-induced AKI for its BUN, and creatinine level was the lowest in all mice with glycerol-induced AKI (Figure 2(C,D)).

### 3.4. Pifithrin-α treatment decreased p53 phosphorylation and oxidative stress-related protein expression and in the renal cortex

**Figure 3.**
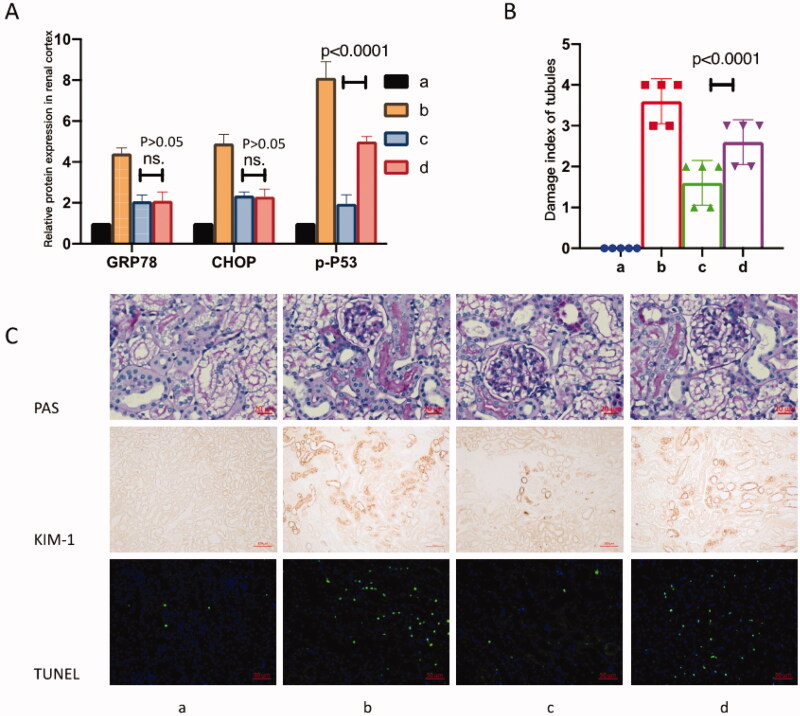
Pifithrin-α treatment ameliorated rhabdomyolysis related AKI. (A) P53 inhibitor pifithrin-α inhibited the renal protein expression of GRP78, CHOP, and p-p53. Notably, pretreatment of pifithrin-α inhibited p-p53 more effectively compared with the pifithrin-α rescue strategy (*p* < 0.001). Nevertheless, pretreatment of pifithrin-α and pifithrin-α rescue therapy showed no difference in affecting GRP78 and CHOP protein levels (*p* > 0.05). (B) P53 inhibitor pifithrin-α ameliorated tubulointerstitial injury in rhabdomyolysis-related AKI. Moreover, pretreatment of pifithrin-α protected PTECs from injury more effectively compared with the pifithrin-α rescue strategy (*p* < 0.001). (C) Representative images of PAS staining, KIM-1 staining, and TUNEL staining of renal slices in four groups. Scale bars were 20 μm, 100 μm, and 50 μm, respectively, as shown on the images.

To investigate the role of p53 activation and oxidative stress in glycerol-induced rhabdomyolysis and AKI affected by pifithrin-α treatment, the relative protein expresses of GRP78, CHOP, and p-p53 were determined in each group (Figure 3(A)). Our results showed that glycerol induction increased the expression of GRP78, CHOP, and p-p53 significantly. Both pretreatments of pifithrin-α and pifithrin-α rescue therapy decreased the expression of GRP78, CHOP, and p-p53. Moreover, pretreatment of pifithrin-α was superior to pifithrin-α rescue therapy in inhibiting p53(Ser15) phosphorylation (*p* < 0.001). It was notable that the pretreatment of pifithrin-α and pifithrin-α rescue therapy showed no difference in affecting GRP78 and CHOP protein levels (*p* > 0.05).

### 3.5. Pifithrin-α treatment ameliorated rhabdomyolysis related renal tubulointerstitial damage

The results showed that glycerol induction incurred severe tubulointerstitial injury and pifithrin-α injection ameliorated tubulointerstitial injury. Especially, compared with pifithrin-α used 4 h after glycerol induction (in Group D), pifithrin-α used 4 h before glycerol induction (in Group C) protected the mice from severe injury more effectively (Figure 3(B), *p* < 0.01). For those nonparametric or mixed data, the Kruskal-Wallis test was used in statistical analyses.

### 3.6. Pifithrin-α treatment ameliorated rhabdomyolysis related renal morphological damage and downregulated the apoptosis pathway

To investigate the role of p53 inhibitor pifithrin-α in glycerol-induced rhabdomyolysis and AKI, the renal morphological damage and potential pathogenic pathway were determined in each group (Figure 3(C)). First, PAS staining revealed massive damages such as inflammatory cells infiltration, renal tubular lumen enlargement, basement membrane of RTECs shedding, and the brush border detachment were ameliorated by pifithrin-α, especially in the pretreatment groups. Secondly, KIM-1 staining confirmed that the RTEC injuries were ameliorated massively by pifithrin-α pretreatment. Finally, the TUNEL assay showed that the renal tubular apoptosis decreased significantly in pifithrin-α pretreated mice, which was consistent with p53 inactivation.

## 4. Discussion

Clinically, AKI is the most common systemic complication of rhabdomyolysis [20]. However, it is not easy to find a thorough description of human or animal kidney specimens in rhabdomyolysis [21]. The underlying mechanisms of AKI triggered by rhabdomyolysis are remarkably complex and diverse. Identification of therapeutic targets would thus depend on revealing common causal pathogenic pathways under distinct conditions. The potential clinical relevance of the present work stems from the similarities between glycerol-induced rhabdomyolysis and crash syndrome associated rhabdomyolysis. Here, we identified p53 activation, oxidative stress enhancement, and increased inflammation played a role in the development of rhabdomyolysis-induced AKI. As expected, pifithrin-α ameliorated glycerol-induced rhabdomyolysis and AKI by directly reducing p53 activation.

Myoglobin exerts a direct toxic effect on the proximal tubule, prompting proximal epithelial cells to secrete inflammatory cytokines [22,23]. Exposure to myoglobin led to overexpression of the inflammasome component and proinflammatory factors. Once into the renal interstitium, myoglobin could directly activate pro-inflammatory cells. Our data showed higher levels of IL-6 in rhabdomyolysis and associated AKI, and this was consistent with glycerol triggered kidney inflammation. Our data also showed simultaneous expression enhancement of GRP78 and CHOP, which represented ER stress-mediated apoptosis pathway enhancement.

To provide evidence for the role of p53 in rhabdomyolysis and associated AKI, we eliminated phosphorylated p53 by using pifithrin-α, and p53 deletion was proved protective in terms of kidney structure and function. Pifithrin-α treatment can lead to an increase in the survival of PTECs in glycerol-treated mice [14]. Beneficial effects of pifithrin-α have been reported in the context of experimental rhabdomyolysis, but conclusions are still controversial [4,11,13–15,24–27]. After glycerol induction, we here observed an acute decrease in renal function, and this was accompanied by severe structural alterations of the kidney, including the injury or apoptosis of PTECs.

P53 can be activated to promote apoptosis during AKI and p53 relative genes were suppressed by pifithrin-α in challenged HK-2 cells [9,24]. The pathologic role of p53 in AKI is complex. Although inhibition of p53 by pifithrin-α or global p53-KO affords protection against cisplatin-induced mice AKI, p53 inhibitors or global p53-KO mice enhanced ischemic induced mice AKI [4,12]. Genotoxic stresses including oncogene activation, hypoxia, and reactive oxygen species in cells, induced DNA damage and then activated p53 expression [12,28]. The renal cell apoptosis, inflammation, cell cycle arrest, and cell death induced by vancomycin were significantly reduced in global p53-KO mice and HK-2 cells treated with pifithrin-α [24]. Previous reports also proved that p53 was involved in renal cell apoptosis, inflammation, cell cycle arrest, and cell death in cisplatin and ischemic induced AKI [9,29]. Our results indicated that pifithrin-α just before the initiation of the aggression could efficiently attenuate the development of AKI.

Heme iron-driven-oxidative stress is one of the dominant mechanisms underlying the glycerol model of rhabdomyolysis and related AKI [30]. Pifithrin-α also can be a free-radical scavenger to inhibit reduction-oxidation (redox) cycling of myoglobin and lipid peroxidation in rhabdomyolysis, thus ameliorating tubule injury. Given that oxidant stress occurs in virtually most forms of AKI, it seems logical that increased free radical formation drives renal p53 accumulation and phosphorylation, culminating in injured renal cells. The use of antioxidants and free-radical scavengers is generally suggested in the treatment or prevention of myoglobinuric AKI, but controlled studies evaluating their efficacy are still lacking. Nevertheless, in HK-2 cells and the mouse model, inhibition of p53 ameliorated glycerol-induced AKI through multi-target regulation [31]. P53 was reported to be involved in kidney injury induced by aristolochic acid, folic acid, and glycerol injection [32]. Our present study suggests the possibility that p53 may be a therapeutic target of AKI caused by glycerol-induced rhabdomyolysis. Given these findings, this study shows that the inhibition of p53 can block glycerol-mediated AKI by using pharmacological instead of genetic inhibitory approaches, which further supports that p53 played a pivotal role in renal tubular injury when challenged with glycerol.

**Figure 4.**
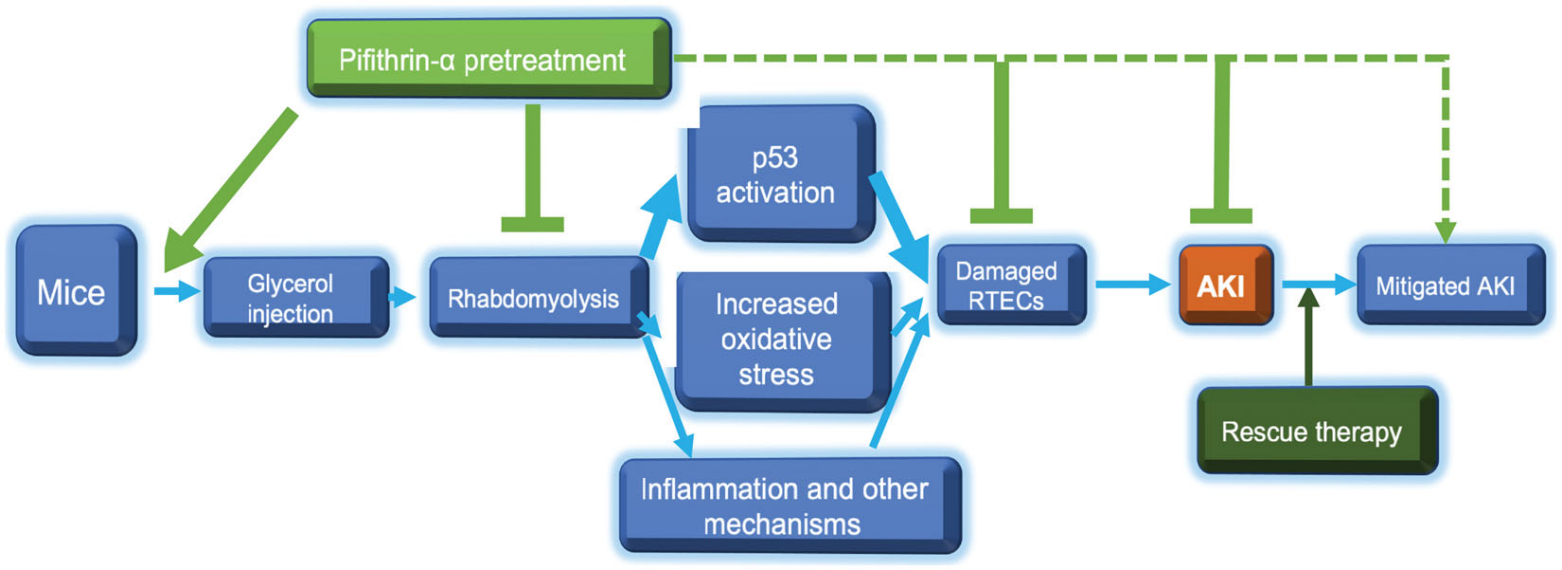
Possible mechanisms of pifithrin-α in ameliorating glycerol-induced rhabdomyolysis and AKI. P53 activation was one of the most crucial events in glycerol-induced rhabdomyolysis and subsequent AKI. Pretreatment of pifithrin-α before AKI inhibited persistent p53 activation and its extent more effectively than the pifithrin-α rescue strategy used after AKI, thus can lead to a relatively favorable outcome.

Taken together, based on our results, we proposed mechanisms of pifithrin-α in ameliorating glycerol-induced rhabdomyolysis and AKI (Figure 4). Mice that underwent intramuscular injection of glycerol developed rhabdomyolysis soon, and consecutive p53 activation, OS activation, inflammation, combined with apoptosis, and other unrecognized mechanisms damaged RTECs, leading to AKI. Pifithrin-α inhibited p53 phosphorylation and OS activation in the kidney and thus ameliorated tubulointerstitial injury and AKI. As pretreatment of pifithrin-α decreased p53 phosphorylation and creatine kinase more effectively and combined with relatively mild rhabdomyolysis and AKI, so it was superior to pifithrin-α rescue therapy in ameliorating rhabdomyolysis and related AKI.
